# Functional Interrogation of Enhancer Connectome Prioritizes Candidate Target Genes at Ovarian Cancer Susceptibility Loci

**DOI:** 10.3389/fgene.2021.646179

**Published:** 2021-03-19

**Authors:** Wei Wang, Fengju Song, Xiangling Feng, Xinlei Chu, Hongji Dai, Jing Tian, Xuan Fang, Fangfang Song, Ben Liu, Lian Li, Xiangchun Li, Yanrui Zhao, Hong Zheng, Kexin Chen

**Affiliations:** ^1^Department of Epidemiology and Biostatistics, National Clinical Research Center for Cancer, Key Laboratory of Molecular Cancer Epidemiology of Tianjin, Tianjin Medical University Cancer Institute and Hospital, Tianjin Medical University, Tianjin, China; ^2^Department of Pharmacology, Tianjin Key Laboratory of Inflammation Biology, School of Basic Medical Sciences, Tianjin Medical University, Tianjin, China; ^3^Department of Gynecological Oncology, Tianjin Medical University Cancer Institute and Hospital, Tianjin Medical University, Tianjin, China; ^4^The Third Department of Breast Cancer, Tianjin Medical University Cancer Institute and Hospital, Tianjin Medical University, Tianjin, China

**Keywords:** H3K27ac-HiChIP, credible variants, CRISPR activation, CRISPR-Cas9 deletion, long-range gene interaction

## Abstract

Identifying causal regulatory variants and their target genes from the majority of non-coding disease-associated genetic loci is the main challenge in post-Genome-Wide Association Studies (GWAS) functional studies. Although chromosome conformation capture (3C) and its derivative technologies have been successfully applied to nominate putative causal genes for non-coding variants, many GWAS target genes have not been identified yet. This study generated a high-resolution contact map from epithelial ovarian cancer (EOC) cells with two H3K27ac-HiChIP libraries and analyzed the underlying gene networks for 15 risk loci identified from the largest EOC GWAS. By combinatory analysis of 4,021 fine-mapped credible variants of EOC GWAS and high-resolution contact map, we obtained 162 target genes that mainly enriched in cancer related pathways. Compared with GTEx eQTL genes in ovarian tissue and annotated proximal genes, 132 HiChIP targets were first identified for EOC causal variants. More than half of the credible variants (CVs) involved interactions that were over 185 kb in distance, indicating that long-range transcriptional regulation is an important mechanism for the function of GWAS variants in EOC. We also found that many HiChIP gene targets showed significantly differential expressions between normal ovarian and EOC tumor samples. We validated one of these targets by manipulating the rs9303542 located region with CRISPR-Cas9 deletion and dCas9-VP64 activation experiments and found altered expression of HOXB7 and HOXB8 at 17q21.32. This study presents a systematic analysis to identify putative target genes for causal variants of EOC, providing an in-depth investigation of the mechanisms of non-coding regulatory variants in the etiology and pathogenesis of ovarian cancer.

## Introduction

Ovarian cancer is one of the most common malignancies in the female reproductive system and accounts for the most deaths from gynecological tumors (Matulonis et al., 2016). Heritable factors play an important role in the development of epithelial ovarian cancer (EOC; Lichtenstein et al., 2000). To date, GWAS have identified approximately 27 loci associated with increased risk of EOC (Song et al., 2009; Bolton et al., 2010; Goode et al., 2010; Bojesen et al., 2013; Permuth-Wey et al., 2013; Pharoah et al., 2013; Kelemen et al., 2015; Kuchenbaecker et al., 2015; Phelan et al., 2017). GWAS identified susceptibility variants are responsible for 6.4% of EOC risk (Phelan et al., 2017). However, most of the GWAS identified risk variants in EOC are located in non-coding regions, which presents challenges when exploration the molecular mechanisms underlying these variants.

Traditionally, the GWAS identified variants were annotated with the nearest gene or biologically relevant proximal genes as the target genes. However, this approach does not consider the three-dimensional conformation of the human genome and its essential role for gene regulation in eukaryotic cells, and these genes may not be true target genes for GWAS identified variants. Recently, chromosome conformation capture (3C) and its derivative technologies (ChIA-PET, capture Hi-C, 4C, etc.) have been successfully used to identify target genes for risk variants and the results revealed that distal rather than the nearest genes are usually the causal targets for the functional variants at GWAS loci. For example, lots of distal target genes for functional GWAS variants have been found through promoter capture Hi-C in colorectal cancer (Jager et al., 2015), cardiovascular diseases (Montefiori et al., 2018), type II diabetes (Miguel-Escalada et al., 2019), and bone mineral density (Chesi et al., 2019). 4C results revealed that vascular diseases susceptibility associated variant rs9349379 was linked with EDN1 which is located 600 kb downstream of the variant (Gupta et al., 2017). HiChIP is a newly developed protein-directed 3C derivative technology with high chromatin conformation capture efficiency and sensitivity (Mumbach et al., 2016). The technology has proven robust in identifying target genes for GWAS identified variants in autoimmune and cardiovascular diseases (Lopez-Isac et al., 2019) and systemic sclerosis (Jeng et al., 2019). In endometrial cancer, the H23K27ac-HiChIP generated chromatin contact map was used to identify target genes for GWAS variants in endometrial cancer (O’Mara et al., 2019). However, there remains a lack of high-resolution genome-scale chromatin contact map in EOC cells to identify target genes for GWAS identified variants.

In this study, we generated a high-resolution H3K27ac-HiChIP contact map from two H23K27ac-HiChIP libraries in EOC cell lines and applied the contact map to identify novel target genes for EOC risk variants. In the largest EOC GWAS study, we fine-mapped 15 risk loci of EOC and created a credible variants (CV) set of 4,021 single nucleotide polymorphisms (SNPs), which covered an estimated 99% of all likely causal variants at the 15 risk loci. After intersecting the CVs with HiChIP generated chromatin contact map, we identified 162 target genes linking to 649 CVs. Most HiChIP targets were newly identified, such as LINC001116, LINC01137, TRIP13, PRC1, and PRC1-AS1. Kyoto Encyclopedia of Genes and Genomes (KEGG) pathway analysis revealed that the target genes were mainly enriched in cancer related pathways, including Wnt and proteoglycans in cancer signaling pathways. In addition, we validated the regulatory relations between rs9303542 and HOXB genes at 17q21.32 with CRISPR-Cas9 deletion and CRISPR activation. Overall, our results provided unique insights into the interaction between risk variants and potential targets with H3K27ac-HiChIP data from EOC cells.

## Results

### A High-Resolution H3K27ac-HiChIP Chromatin Contact Map in EOC Cell Lines

To generate a high-resolution genome-wide long-range chromatin contact map in EOC, we performed H3K27ac-HiChIP in two EOC cell lines, OVCA432 and SKOV3. We sequenced the HiChIP library of SKOV3 to 400 million reads and OVCA432 to 200 million reads. We used the HiC-pro pipeline for quality control (QC) with default settings (Servant et al., 2015). After removing the unmapped reads and duplicated interaction pairs, 267,408,761 valid interaction pairs were identified in SKOV3, in which 84% were classified as unique valid interactions. In all valid interaction pairs, 55% were *cis* long-range interactions (>20,000 bp), 18% were *cis* short-range interactions (<20,000 bp) and 11% were *trans* interactions (Supplementary Figure 1A). Among the 137,387,655 valid interaction pairs identified in OVCA432, 83% were unique valid interaction pairs. The percentage of *cis* long-range interactions, *cis* short-range interactions, and *trans* contacts were 52, 17, and 14%, respectively (Supplementary Figure 1B). Detailed QC results are listed in Supplementary Table 1. Both libraries fulfilled the requirement for a high-quality HiChIP library. We used the Fit-HiChIP pipeline (Bhattacharyya et al., 2019) to call significant interactions that were ranged from 20 to 2,000 kb in distance. In total, we identified 161,309 significant loops with a median distance of 145 kb (mean distance: 258,124 bp) in SKOV3 (Supplementary Figure 1C) and 113,357 significant interactions with a median distance of 110 kb (mean distance: 163,129 bp) in OVCA432 (Supplementary Figure 1D).

The distal regulatory elements usually physically interacted with the gene promoter to regulate its expression. Therefore, we selected promoter-associated loops that looped with a promoter region in at least one end. We separated the promoter-associated loops into four categories, including promoter–promoter, promoter–distal intergenic, promoter–intron, and promoter–others (UTR, gene downstream, and exon) (Supplementary Figure 2A). The results suggested that about half of the promoter-associated loops were either promoter to distal intergenic or promoter to intron interactions. This result reflects the distribution of H3K27ac marked enhancers in the human genome. Approximately 20% of the promoter-associated loops were promoter–promoter loops, indicating that promoters could contact and thus influence each other under transcription regulation in EOC development. About one-third of promoter-associated loops overlapped between the two EOC cell lines, indicating there were shared mechanisms for long-range regulation in these cells (Supplementary Figure 2B).

To explore the transcription factors that mediate long-range gene regulation in EOC, we conducted HOMER (Heinz et al., 2010) analysis on ATAC-seq marked sequence in non-promoter end of promoter-associated loops. As expected, CCCTC-binding factor (CTCF) showed enrichment of the non-promoter end in promoter-associated loops, which was consistent with its function in mediating long-distance chromatin contact (Li et al., 2020). The results also suggested that AP-1 transcription factor complex members, such as JUNB, FRA1, FRA2, and FOSL2, were the most enriched motifs in both EOC cells (Supplementary Figures 2C,D). This data indicates that AP-1 transcription factors may act as a master organizer for the long-range gene regulation in EOC.

### H3K27ac-HiChIP Interactions Linked the Likely Causal SNPs of EOC to Their Target Genes

**FIGURE 1:**
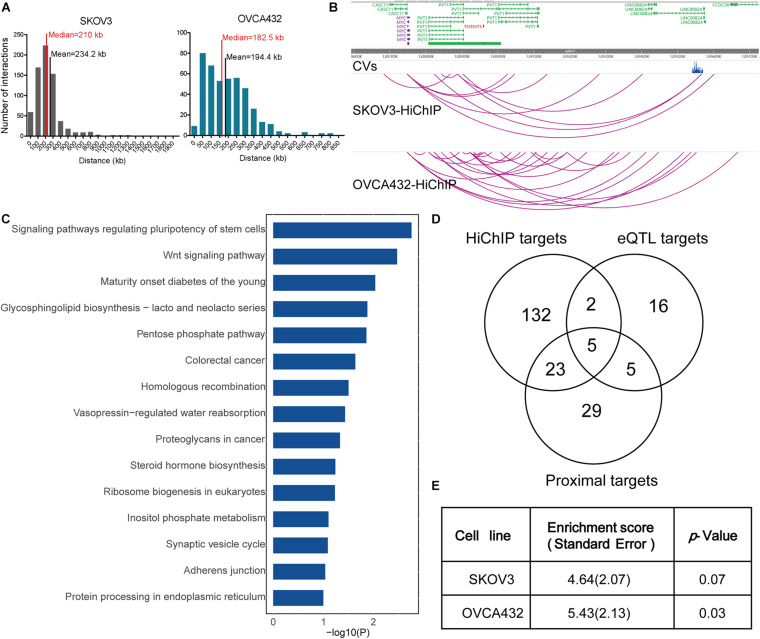
HiChIP identified gene targets for GWAS loci of EOC. **(A)** Distribution of the distances spanning each CVs involved in the HiChIP loop in SKOV3 and OVCA432. The red line indicates the median distance and the black line indicates the mean distance. **(B)** Examples of MYC and PVT1 looping to EOC GWAS CVs at 8q21.24 loci. **(C)** KEGG pathway analysis for HiChIP identified gene targets looping to GWAS CVs. **(D)** Venn plot displaying the number of HiChIP targets, eQTL targets, and proximal targets of GWAS CVs of EOC. **(E)** SLDSC enrichment analysis for HiChIP loops identified from SKOV3 and OVCA432 cells.

To understand the gene regulation mechanisms underlying EOC GWAS variants, we focused our analysis on 15 genomic regions identified from the largest GWAS study in EOC. We fine-mapped the 15 risk loci with a Bayesian approach and generated a credible set that covered a 99% estimate of all likely causal variants at each locus. In total, we got 4,021 credible variants (Supplementary Table 2). After intersecting the CVs with significant HiChIP loops, we achieved 162 putative target genes for 649 CVs that fell in the loops (Table 1). The 649 CVs located in the significant HiChIP loops were defined as HiChIP-CVs, and the genes located on the other end of the loops, which have CVs at one end, were defined as HiChIP target genes. The distance between the HiChIP-CVs to gene targets ranged from 20 to 1,810 kb. The median distance between the causal variants and their targets is 210 kb in SKOV3 and 182 kb in OVCA432 (Figure 1A). For example, the HiChIP results revealed that MYC and PVT1 were the most likely targets for CVs at 8q24.21 (Pomerantz et al., 2009; Ahmadiyeh et al., 2010; Sotelo et al., 2010; Grampp et al., 2016; Lancho and Herranz, 2018), which was 815 kb away from their linked variants (Figure 1B). Detailed information on HiChIP-CVs and target genes is listed in Supplementary Tables 3, 4.

**TABLE 1:** HiChIP target genes at EOC risk loci.

**Locus**	**CV Counts**	**HiChIP Target Genes**
1p34.3	5	RP11-109P14.10, MTF1, INPP5B, GNL2*, LINC01137, ZC3H12A
2q31.1	17	LINC01116*, LINC01117, HOXD1, AC079305.8, MIR4444-2, HNRNPA3
3q25.31	54	PLCH1, AC104472.1, C3orf33, SSR3, TIPARP, TIPARP-AS1*, LEKR1, LINC00880, RP11-6F2.5, LINC00881, CCNL1, RNA5SP146, RP11-550I24.2, LINC00886*, PA2G4P4
5p15.33	1	TRIP13*
8q24.21	32	CASC8, CASC11, RP11-419K12.1, RP11-89M16.1, MYC, PVT1
9p22.2	11	BNC2*
9q34.2	35	ABO, RP11-430N14.4, RALGDS, SURF4*, C9orf96, REXO4, ADAMTS13
10p12.31	5	RP11-275N1.1, NEBL-AS1, NEBL*, MLLT10*
15q26.1	28	RN7SL346P, SEMA4B*, RP11-154B12.3, IQGAP1*, HDDC3*, RP11-387D10.2, RP11-387D10.3, UNC45A*, AC068831.3, RCCD1*, AC068831.6, VPS33B, AC068831.10, PRC1*, PRC1-AS1, AC068831.11, AC068831.12, RP11-661P17.1, CTD-2313J17.1, FAM174B*
17q12	10	GGNBP2,DHRS11*, SYNRG*, DDX52, RP11-697E22.1, RP11-697E22.2, RP11-697E22.3, HNF1B, YWHAEP7, AC124789.1, ARHGAP23*
17q21.31	244	AC002117.1, HEXIM2*, CTD-2020K17.1, FMNL1*, DND1P1, RP11-259G18.1, KANSL1*, NSF, WNT3, ARL17B
17q21.32	96	SKAP1*, RP11-456D7.1, RNU6-1152P, HOXB3*, HOXB-AS3, HOXB-AS2, HOXB4*, MIR10A, HOXB7*, HOXB8*, HOXB9, HOXB6, HOXB5, HOXB2, HOXB-AS1, Y_RNA, COPZ2*, CBX1, SNX11*, MIR1203, RP11-357H14.17, RP11-433M22.2, HOXB-AS4, MIR196A1
19p13.11	15	CTC-429P9.4, SMIM7*, TMEM38A, HAUS8*, MYO9B, USE1, OCEL1, NR2F6*, AC010646.3, USHBP1, ANO8*, GTPBP3*, CTD-3131K8.2, PGLS*, ABHD8, DDA1*, PLVAP, CTD-2521M24.9, BST2, MVB12A*, CTD-2521M24.6, CTD-2521M24.8, CTD-2521M24.5
22q12.1	96	PITPNB, TTC28-AS1, MIR3199-2, TTC28, CHEK2, CCDC117, CTA-292E10.6, XBP1, HSCB, RN7SL162P, ZNRF3-AS1, ZNRF3, RHBDD3, EWSR1*, MIAT*, CTA-373H7.7, CTA-211A9.5, CTA-292E10.7

Underlined loci or genes are shown in HiChIP results of both EOC cell lines.

*Genes are differentially expressed between normal samples and tumor samples of EOC in at least one of GSE18520, GSE27651, and GSE54388 dataset (Fold change ≥ 2 and adjust *p*-value < 0.01).

We performed KEGG pathway analysis for the HiChIP target genes and found that these target genes were enriched in some cancer related pathways, like signaling pathways regulating pluripotency of stem cells, homologous recombination, Wnt signaling pathway, and proteoglycans in cancer (Figure 1C). To further test the relevance of the HiChIP target genes in tumorigenesis of EOC, we compared the HiChIP targets with differential expressed genes between tumor and normal ovarian tissue from three GEO datasets [GSE18520 (Mok et al., 2009), GSE27651 (King et al., 2011), and GSE54388 (Yeung et al., 2017)]. We found that 24% (39 out of 162) HiChIP target genes had significant difference in expression between normal and tumor ovary samples (Table 1). Moreover, the 162 HiChIP target genes showed a higher enrichment score in tumor ovarian samples than normal ovarian samples in all three GEO datasets (Supplementary Figure 3A). The difference is more significant when only the differentially expressed HiChIP target genes were considered (Supplementary Figure 3B). These results indicated that HiChIP identified targets were involved in EOC tumorigenesis.

Next, we compared the expression Quantitative Trait Loci (eQTL) targets and proximal genes of GWAS CVs with HiChIP targets. We collected 28 eQTL target genes from the Genotype-Tissue Expression (GTEx) expression of ovary tissue and 62 proximal genes from the annotation of the CVs. Compared with eQTL and proximal targets, the HiChIP found more target genes, with 5.4-fold to the eQTL targets and 2.5-fold to the proximal targets (Figure 1D). Among these target genes, 7 HiChIP targets overlapped with eQTL targets, and 28 targets overlapped with proximal targets. Five genes were identified in all three target sets, including ABO, DND1P1, KANSL1, NSF, and PRC1-AS1. The eQTL and proximal target genes were listed in Supplementary Table 5.

To find out whether HiChIP loops were enriched with GWAS signals of EOC, we applied the stratified linkage disequilibrium score regression (SLDSR) method to quantify the enrichment of GWAS signals at the HiChIP loops. The results suggested strong enrichment of EOC risk variants in the HiChIP loops when compared to the random genomic variants at the 15 risk loci from the largest EOC GWAS study (Figure 1E). This result confirmed that GWAS signals prefer to reside in regulatory genomic regions and affect target genes through long-range regulation.

### Functional Validation of Causal Variant Effect on HOXB Genes at 17q21.32 Loci

**FIGURE 2:**
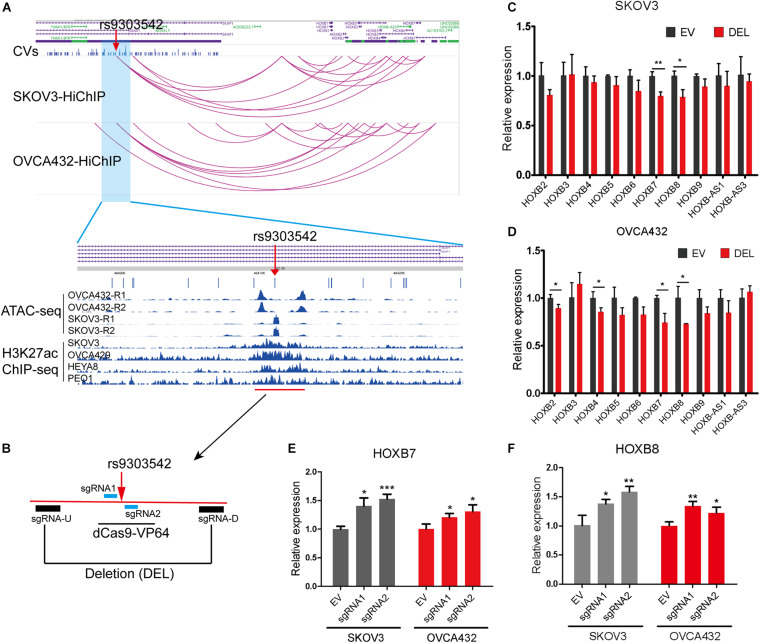
Validation of regulatory relations between rs9303542 enhancer region and HOXB genes. **(A)** Interaction profiles of rs9303542 and HOXB genes at 17q21.32 and ATAC-seq and H3K27ac-ChIP signal enrichment at the rs9303542 region. **(B)** A schematic representation elucidating the design for CRISPR-Cas9 deletion and dCas9-VP64 activation experiments. sgRNA-U and sgRNA-D were cloned in px459v2 respectively and then cotransfected into the indicated cells to delete the 2000bp rs9303542 enhancer region. The sgRNA1/2 were separately cloned into MS2-gRNA-hU6 expression vector and then transfected into dCas9-VP64 stable expression SKOV3 and OVCA432 cells. **(C,D)** qPCR was used to detect the expression of HOXB genes between rs9303542 deleted (DEL) and vector control (EV) cells in SKOV3 **(C)** and OVCA432 **(D)**. The expression of HOXB1, HOXB-AS2, and HOXB-AS4 was too low to detect in both cell lines. **(E,F)** qPCR was used to compare the expression of HOXB7 **(E)** and HOXB8 **(F)** after sgRNA1/2 transfected (sgRNA1, sgRNA2) and empty vector transfected (EV) cells with dCas9-VP64 stable expression. Error bars, SD. ns: not significant, *p < 0.05, **p < 0.01, ***p < 0.001 as determined by an unpaired, two-tailed Student’s *t*-test.

To further identify the likely causal variants of these 649 HiChIP-CVs, we intersected the 649 HiChIP-CVs with ATAC-seq peaks from SKOV3 and OVCA432 cells. This narrowed the HiChIP-CVs to 39 SNPs that featured ATAC-seq peaks in both cells (Supplementary Table 6). Next, we focused on 17q21.32 loci, as this loci presented the most HiChIP targets and likely causal variants. At 17q21.32 loci, the index SNP rs9303542 located genomic region interacted with HOXB genes from the HiChIP results in both cells, which implied a regulatory element for HOXB genes at this location. Many studies have found that HOXB cluster members are involved in the tumorigenesis or progression of ovarian cancer, including HOXB2 (Yu and Pan, 2020), HOXB3 (Miller et al., 2018), HOXB4 (Li et al., 2018), HOXB7 (Chen et al., 2020), and HOXB8 (Stavnes et al., 2013). Moreover, ATAC-seq and H3K27ac ChIP results from various EOC cell lines revealed that rs9303542 located in open chromatin and the H3K27ac marked enhancer region (Figure 2A). To validate the long-range regulation between the rs9303542-containing enhancer and HOXB genes, we deleted an approximate 2 kb H3K27ac marked enhancer region around rs9303542 in OVCA432 and SKOV3 cells (Figure 2B) and measured the expression changes of HOXB genes after rs9303542 deletion. Because rs9303542 is located in the intron of SKAP1, the expression of SKAP1 was also checked. As expected, HOXB7 and HOXB8 showed a significant decrease after rs9303542 deletion in both cells while SKAP1 (Supplementary Figures 4A,B) and many other HOXB genes showed a moderate decrease in expression after rs9303542 deletion (Figures 2C,D). To further confirm whether rs9303542 was located in the enhancer region and directly regulated the expression of HOXB7 and HOXB8, we designed two sgRNAs (sgRNA1: 38 bp upstream of rs9303542, sgRNA2: 25 bp downstream of rs9303542) around the rs9303542 and expressed the sgRNAs in dCas9-VP64 stable expression cells (Figure 2B). The results revealed that the expression of HOXB7 and HOXB8 were significantly increased in sgRNAs expressed dCas9-VP64 cells (Figures 2E,F), but no changes were observed in SKAP1 expression (Supplementary Figure 4C). These results indicated that the rs9303542-containing enhancer region had a direct role in regulating HOXB gene expression, especially for HOXB7 and HOXB8.

**FIGURE 3:**
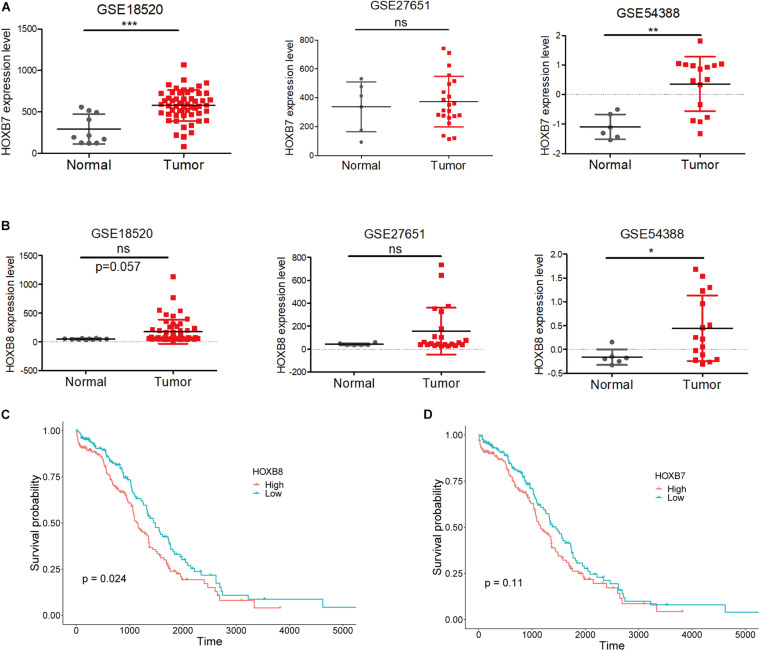
Expression and survival analysis for HOXB7 and HOXB8. **(A,B)** Expression levels of HOXB7 **(A)** and HOXB8 **(B)** in three GEO datasets. **(C,D)** Overall survival analysis for EOC patients in the TCGA database was based on the expression of HOXB8 **(C)** and HOXB7 **(D)** expression from TCGA data. Median expression was used to stratify the high and low expression groups. ns: not significant, *p < 0.05, **p < 0.01, ***p < 0.001 as determined by an unpaired, two-tailed Student’s *t*-test.

Moreover, expression analysis between normal and tumor ovarian samples from three GEO datasets (GSES18520, GSE27651, and GSE54388) showed that HOXB7 expression was significantly increased in EOC tumors as two out of three GSE datasets showed significantly upregulated HOXB7 expression in EOC tumor samples (Figure 3A). At the same time, HOXB8 also showed upregulation in EOC tumor samples in two GSE datasets with one dataset reached a marginal significant *p*-value (Figure 3B). Overall survival analysis revealed that increased expression of HOXB8 was associated with a short survival time (Figure 3C). The expression of HOXB7 is also associated with the overall survival of EOC patients with a marginal significant *p* value (Figure 3D). These results indicated that HOXB7 and HOXB8 may play a role in the tumorigenesis of EOC and that rs9303542 was a likely causal variant at the 17q21.32 loci through regulating HOXB7 and HOXB8 expression to affect the tumor development of EOC.

## Discussion

Three-dimension chromosome architecture can bring distal regulatory elements like enhancers into close contact with target genes to *cis* regulate gene expression through binding transcription factors. Recent studies have found that some GWAS SNPs in distal regulatory elements can affect the binding affinity of transcription factors due to the different genotypes present in the binding motifs of transcription factors (Miller et al., 2018; Yu and Pan, 2020). Understanding the regulatory landscape of non-coding variants at GWAS identified risk loci is the main obstacle for current post-GWAS studies. In the present study, we generated a high-resolution contact map from two H3K27ac-HiChIP libraries in EOC cells. With the high-resolution interaction map, we identified 162 target genes for non-coding variants at 14 EOC risk loci. Most of the HiChIP targets are distal genes. Many HiChIP gene targets show differential expression between normal ovarian and tumor ovarian samples. The HiChIP targets were enriched in some cancer related pathways as well. These results indicated that HiChIP identified targets could be disease causal genes that are involved in the tumorigenesis of EOC. Lots of HiChIP targets were first identified as compared with eQTL and proximal targets. Stratified linkage disequilibrium score regression (SLDSC) enrichment analysis revealed that GWAS variants of EOC showed higher enrichment in HiChIP loops than random genomic variants, indicating the heritable relevance of HiChIP data from the two EOC cell lines and high enrichment of GWAS variants in regulatory elements in the genome.

The current available relevant chromatin contact data for EOC is generated with HiC from ovary tissues (Schmitt et al., 2016), since HiC was designed to explore all chromatin contact and thus needed very high sequencing depth to reach a high resolution, this HiC data was usually applied to detect the higher chromosome structure like TAD in EOC but not to explore the target genes for GWAS variants due to a low resolution. Recently, O’Mara et al. (2019) analyzed the H23K27ac-HiChIP identified target genes at shared GWAS risk loci of endometrial cancer and ovarian cancer with HiChIP data from a normal ovarian cell line (Glubb et al., 2020), normal and tumor endometrial cell lines (O’Mara et al., 2019). We compared the HiChIP identified target genes in endometrial cells and EOC at 1p34.3, 8q24, and 17q21.32, which were risk loci for both cancers. Lots of targets were the same between the two cancer types, like GNL2 at 1p34.3, PVT1 at 8q24, and HOXB genes at 17q21.32, indicating some common genetics shared between these two gynecologic tumors. However, different target genes were observed as well, like CASC8, CASC11, and LINC01137 were only detected in EOC cells, indicating the different roles of these genes in tumorigenesis between endometrial cancer and EOC. The chromosome conformation is always changed with the epigenetic changes during tumorigenesis which resulted in the dysregulated gene regulation in tumor development (Taberlay et al., 2016; Li et al., 2019). Thus, long-range interactions might be different between normal and tumor ovarian cells. We compared the target genes identified from normal ovarian cells by the O’Mara group and ovarian tumor cells from our study and found some common targets like ABO at 9q34.2, MYC at 8q24.21, and METTL10 at 10p12.31. We also found different HiChIP target genes identified between normal tumor ovarian cell lines, like CASC10, MIR1915, and SKIDA1 were only identified for GWAS variants in normal ovarian cell lines while NEBL-AS1 and NEBL were identified in tumor cell lines, indicating that different long-range regulation patterns existed between normal ovarian and EOC tumor cells.

HOXB genes are important housekeeping genes and many HOXB cluster members have been revealed to play important roles in tumorigenesis (Bhatlekar et al., 2014). A previous study used HiC and found that rs9303542 is located in the same TAD domain as HOXB genes (Phelan et al., 2017). Our HiChIP results, as well as the HiChIP results from endometrial cells, revealed direct links between rs9303542 and HOXB genes. We found rs9303542 located in an enhancer region with the ATAC-seq and H3K27ac signals. Moreover, manipulating the rs9303542 region could change the expression of HOXB genes especially for HOXB7 and HOXB8. We also observed upregulated expression of HOXB7 and HOXB8 in EOC tumor samples as well as the association between HOXB7 and HOXB8 expression and overall survival for EOC patients, the results are consistent with previous findings for HOXB7 and HOXB8 in various human cancers including ovarian cancer (Errico et al., 2016; Monterisi et al., 2018; Chen et al., 2020; Ying et al., 2020). Even though, with strong evidence from HiChIP results and validation results, no significant eQTL associations were found between rs9303542 and the expression of HOXB7 (Supplementary Figure 5A) or HOXB8 (Supplementary Figure 5B). This is probably due to the small sample size for rs9303542 minor allele (GG) (*n* = 18) in the analysis. These results reveal that rs9303542 might be a causal variant at 17q21.32 and affected tumorigenesis of EOC through long-distance regulation of HOXB7 and HOXB8 expression. However, further experiments are needed to prove the genotype-specific regulation of rs9303542 on HOXB7 and HOXB8 expression as well as the oncogenic roles of these two genes on the tumorigenesis of EOC.

We also found that the rs9303542 region can only slightly change the expression of HOXB genes, as revealed from the little fold changes in expression of HOXB genes in rs9303542 deletion and dCas9-VP64 activation experiments. At the same time, we also observed that rs9303542 linkage variants rs8067953 (LD = 0.99 to rs9303542) at the same risk loci also targeted HOXB genes, which were also located in an enhancer region (Supplementary Figure 5C). Therefore, we speculated that the composite effect might exist for linkage GWAS variants to regulate the targeted genes corporately to largely affect tumor development.

In summary, this study has identified candidate gene targets for GWAS variants of EOC and explored some previously unknown links between risk variants and distal targets, which provides some insightful views in exploring the underlying genetic basis of ovarian cancer development.

## Materials and Methods

### Cell Culture

Human SKOV3 ovarian cancer cells were obtained from the American Type Culture Collection (ATCC). The human OVCA432 ovarian cancer cells were gifts from Dr. Wei Zhang of The University of Texas MD Anderson Cancer Center in Houston, TX, and preserved in our lab. SKOV3 and OVCA432 were cultured in complete DMEM medium supplemented with 10% FBS, 100 μg/mL penicillin, and 100 μg/mL streptomycin. All cells were cultivated in a 37°C humid incubator with 5% CO_2_.

### H3K27ac-HiChIP Library Generation

H3K27ac-HiChIP was performed mainly following the procedures of Mumbach et al. (2016) with little modifications. Briefly, 15 million SKOV3 and OVCA432 cells were harvested and crosslinked with 1% formaldehyde in PBS at room temperature for 10 min, then quenched with 125 mM Glycine on ice for 5 min. Next, crosslinked cells were lysed in Hi-C lysis buffer and nuclei were extracted and digested with Mbo1 restriction enzyme (NEB, R0147) for 2 h. After digestion, nuclei were resuspended in NEB buffer supplemented with DNA polymerase 1, Large (Klenow) fragment (NEB, M0210) to fill in the restriction fragment overhangs with biotin labeled dATP for 1 h. Proximal ligation was performed with T4 DNA ligase for 4 h at 16°C and nuclei were harvested. Palleted nuclei were transferred to Covaris S220 sonicator to shear chromatin with the same procedure as Mumbach et al. (2016) For each sample, sheared chromatin was incubated overnight with 7.5 μL of H3K27ac antibody (Abcam, ab4729) and then Protein A beads were used to capture H3K27Ac-associated chromatin, the whole H3K27ac-ChIP process was performed with the Pierce^TM^ Magnetic ChIP kit (#26157), the final DNA was eluted with 10 μL water. Eluted DNA was sent for DNA biotin pull-down with streptavidin beads. Fifty microgram of DNA was used to PCR amplification and then page purigy was performed to select a size range of 300–700 bp products. Final purified PCR products were quantified with qPCR against Illumina primers and sent for sequencing.

### HiChIP Data Analysis

HiChIP paired-end reads were aligned to the hg19 human genome using the HiC-Pro pipeline (Servant et al., 2015) with default settings to remove duplicate reads, assign reads to *Mbo*I restriction fragments, filter for valid interactions, and generate binned interaction matrices. Fit-HiChIP pipeline was applied to HiC-Pro 5k base pair resolution matrices to call the significant loops (Bhattacharyya et al., 2019). Chromatin interactions were filtered within a range from a minimum distance of 20 kb to a maximum of 2 Mb.

### Target Gene Identification With HiChiP Data and eQTL

We fine-mapped the 15 EOC GWAS loci identified from the largest EOC GEAS study with a standard Bayesian approach. And then the 99% CVs were defined to cover the 99% estimate of all functional variants at each risk loci. Then the CVs were intersected with significant loops called from the Fit-HiChIP pipeline with bedtools. If the CVs were located at one end of the loop, the genes annotated at the other end of the loop were identified as a HiChIP target gene for the CV. Identification of eQTL targets was undertaken using public eQTL databases, including ovarian tissues from GTEx (data release v7)^1^ (Consortium, 2013) and ovarian tumor samples from pancan QTL^2^ (Gong et al., 2018).

### Stratified LD Score Regression Analysis

Stratified LD score regression (Bulik-Sullivan et al., 2015; Finucane et al., 2015) was used to quantify the enrichment score of EOC GWAS risk variants in promoter-associated loops of EOC as O’Mama used before (O’Mara et al., 2019). In brief, stratified LD score regression compared the enrichment of genetic heritability associated with EOC risk locating in the HiChIP loops with total genetic variants falling in the HiChIP loops. The enrichment scores for the promoter-associated loops of the two EOC cell lines were calculated separately, conditioned on a baseline model (Finucane et al., 2015) as background normalization. The HapMap3 variants and Genome 1000 Project variant of the European population were used as a reference in LD calculation.

### CRISPR Deletion

To delete enhancer fragment containing rs9303542 (2 kb) in SKOV3 and OVCA432 cells, the px459v2 vector containing sgRNA-U and sgRNA-D (1.5 μg each) or px459v2 empty vector (3 μg) was cotransfected into target cells by using Lipofectamine3000 transfection reagent (Invitrogen). After selection with puromycin (2 μg/mL for SKOV3 and 20 ug/ml for OVCA432) for 3 days, the remaining cells were cultured for another 3 days. Then RNA was extracted with TRIZOL, and qPCR was performed to detect the expression of HOXB genes. All qPCR primers and sgRNA oligos used are listed in Supplementary Table 7.

### CRISPR Activation

To activate the enhancer activity surrounding rs9303542 in OVCA432 and SKOV3 cells, two gRNAs sgRNA-1/2 around rs9303542 were designed and cloned into the MS2-gRNA-hU6 vector. Then, gRNAs were transfected into dCas9-VP64 stable expression OVCA432 and SKOV3 cells by using Lipofectamine 3000 transfection reagent (Invitrogen). Forty-eight hours after transfection, RNAs were extracted and reverse transcribed. Then qPCR was performed to test the expression for HOXB7 and HOXB8.

### HiChIP Data Visualization

The FitHiChip called significant loops after merging were visualized with WashU Epigenome Browser^3^. The ATAC-seq for SKOV3 and OVCA432 were generated by our own and H3K27ac-ChIP data of SKOV3, OVCA429, HEYA8, and POE1 EOC cell lines were obtained from GEO datasets (Chung et al., 2019).

### Gene Enrichment Analysis

Single-sample gene-set enrichment analysis (ssGSEA) was conducted through the GSVA package and its ssGSEA method^4^, enrichment score of 162 HiChIP target genes or 39 differentially expressed HiChIP target genes in each sample from three GEO datasets were calculated. Boxplot was used to show the difference in enrichment scores between normal and tumor ovarian samples.

## Data Availability Statement

The datasets presented in this study can be found in online repositories. The names of the repository/repositories and accession number(s) can be found below: National Genomics Data Center (NGDC) (https://bigd.big.ac.cn/) under accession number HRA000658.

## Author Contributions

KC, FeS, and WW designed the research. WW performed the experiments and wrote the manuscript. XiF performed the data analyses. JT, XuF, XC, and HD contributed significantly to the manuscript preparation. FaS, BL, LL, XL, YZ, and HZ helped with constructive discussions. All authors contributed to the article and approved the submitted version.

## Conflict of Interest

The authors declare that the research was conducted in the absence of any commercial or financial relationships that could be construed as a potential conflict of interest.

## Footnotes

1 http://gtexportal.org

2 http://gong_lab.hzau.edu.cn/PancanQTL/

3 https://epigenomegateway.wustl.edu/

4 http://www.bioconductor.org

## Supplementary Material

Supplementary Figure 1 QC of HiChIP results with HiC-pro pipeline and loop calling results with Fit-HiChIP pipeline.

Supplementary Figure 2 General features of Fit-HiChIP identified loops in EOC.

Supplementary Figure 3 Enrichment score of HiChIP target genes calculated by ssGSEA between normal and tumor ovarian samples in three GEO datasets.

Supplementary Figure 4 Expression of SKAP1 expression in SKOV3 and OVCA432 cells after modulating rs9303542 enhancer region.

Supplementary Figure 5 eQTL analysis of HOXB7 and HOXB8 as well as loop profiles of rs8067953.

Supplementary Table 1 HiC-Pro QC results.

Supplementary Table 2 Ninety-nine percent of CVs used in our study.

Supplementary Table 3 Detailed loop information of CVs to target genes in SKOV3.

Supplementary Table 4 Detailed loop information of CVs to target genes in OVCA432.

Supplementary Table 5 Proximal targets and eQTL targets of HiChIP-CVs.

Supplementary Table 6 Detailed information of 39 HiChIP-CVs with ATAC-seq signals in both SKOV3 and OVCA432 cells.

Supplementary Table 7 The primer list used in the study.

## References

[B1] Ahmadiyeh N. Pomerantz M. M. Grisanzio C. Herman P. Jia L. Almendro V. (2010). 8q24 prostate, breast, and colon cancer risk loci show tissue-specific long-range interaction with MYC. *Proc. Natl. Acad. Sci. U.S.A.* 107 9742–9746. 10.1073/pnas.0910668107 20453196 PMC2906844

[B2] Bhatlekar S. Fields J. Z. Boman B. M. (2014). HOX genes and their role in the development of human cancers. *J. Mol. Med.* 92 811–823. 10.1007/s00109-014-1181-y 24996520

[B3] Bhattacharyya S. Chandra V. Vijayanand P. Ay F. (2019). Identification of significant chromatin contacts from HiChIP data by FitHiChIP. *Nat. Commun.* 10:4221. 10.1038/s41467-019-11950-y 31530818 PMC6748947

[B4] Bojesen S. E. Pooley K. A. Johnatty S. E. Beesley J. Michailidou K. Tyrer J. P. (2013). Multiple independent variants at the TERT locus are associated with telomere length and risks of breast and ovarian cancer. *Nat. Genet.* 45 371–384, 384e1–e2. 10.1038/ng.2566 23535731 PMC3670748

[B5] Bolton K. L. Tyrer J. Song H. Ramus S. J. Notaridou M. Jones C. (2010). Common variants at 19p13 are associated with susceptibility to ovarian cancer. *Nat. Genet.* 42 880–884. 10.1038/ng.666 20852633 PMC3125495

[B6] Bulik-Sullivan B. K. Loh P. R. Finucane H. K. Ripke S. Yang J. Schizophrenia Working Group of the Psychiatric Genomics Consortium (2015). LD Score regression distinguishes confounding from polygenicity in genome-wide association studies. *Nat. Genet.* 47 291–295. 10.1038/ng.3211 25642630 PMC4495769

[B7] Chen Y. Zhao X. H. Zhang D. D. Zhao Y. (2020). MiR-513a-3p inhibits EMT mediated by HOXB7 and promotes sensitivity to cisplatin in ovarian cancer cells. *Eur. Rev. Med. Pharmacol. Sci.* 24 10391–10402. 33155195 10.26355/eurrev_202010_23389

[B8] Chesi A. Wagley Y. Johnson M. E. Manduchi E. Su C. Lu S. (2019). Genome-scale capture C promoter interactions implicate effector genes at GWAS loci for bone mineral density. *Nat. Commun.* 10:1260. 10.1038/s41467-019-09302-x 30890710 PMC6425012

[B9] Chung V. Y. Tan T. Z. Ye J. Huang R. L. Lai H. C. Kappei D. (2019). The role of GRHL2 and epigenetic remodeling in epithelial-mesenchymal plasticity in ovarian cancer cells. *Commun. Biol.* 2:272. 10.1038/s42003-019-0506-3 31372511 PMC6656769

[B10] Consortium G. T. (2013). The genotype-tissue expression (GTEx) project. *Nat. Genet.* 45 580–585. 10.1038/ng.2653 23715323 PMC4010069

[B11] Errico M. C. Jin K. Sukumar S. Care A. (2016). The widening sphere of influence of HOXB7 in solid tumors. *Cancer Res.* 76 2857–2862. 10.1158/0008-5472.CAN-15-3444 27197229 PMC4874556

[B12] Finucane H. K. Bulik-Sullivan B. Gusev A. Trynka G. Reshef Y. Loh P. R. (2015). Partitioning heritability by functional annotation using genome-wide association summary statistics. *Nat. Genet.* 47 1228–1235. 10.1038/ng.3404 26414678 PMC4626285

[B13] Glubb D. M. Thompson D. J. Aben K. K. Alsulimani A. Amant F. Annibali D. (2020). Cross-cancer genome-wide association study of endometrial cancer and epithelial ovarian cancer identifies genetic risk regions associated with risk of both cancers. *Cancer Epidemiol. Biomarkers. Prev.* 30 217–228. 10.1158/1055-9965.EPI-20-0739 33144283

[B14] Gong J. Mei S. Liu C. Xiang Y. Ye Y. Zhang Z. (2018). PancanQTL: systematic identification of cis-eQTLs and trans-eQTLs in 33 cancer types. *Nucleic Acids Res.* 46 D971–D976. 10.1093/nar/gkx861 29036324 PMC5753226

[B15] Goode E. L. Chenevix-Trench G. Song H. Ramus S. J. Notaridou M. Lawrenson K. (2010). A genome-wide association study identifies susceptibility loci for ovarian cancer at 2q31 and 8q24. *Nat. Genet.* 42 874–879. 10.1038/ng.668 20852632 PMC3020231

[B16] Grampp S. Platt J. L. Lauer V. Salama R. Kranz F. Neumann V. K. (2016). Genetic variation at the 8q24.21 renal cancer susceptibility locus affects HIF binding to a MYC enhancer. *Nat. Commun.* 7:13183. 10.1038/ncomms13183 27774982 PMC5079059

[B17] Gupta R. M. Hadaya J. Trehan A. Zekavat S. M. Roselli C. Klarin D. (2017). A genetic variant associated with five vascular diseases is a distal regulator of endothelin-1 gene expression. *Cell* 170 522.e15–533.e15. 10.1016/j.cell.2017.06.049 28753427 PMC5785707

[B18] Heinz S. Benner C. Spann N. Bertolino E. Lin Y. C. Laslo P. (2010). ., Simple combinations of lineage-determining transcription factors prime cis-regulatory elements required for macrophage and B cell identities. *Mol. Cell* 38 576–589. 10.1016/j.molcel.2010.05.004 20513432 PMC2898526

[B19] Jager R. Migliorini G. Henrion M. Kandaswamy R. Speedy H. E. Heindl A. (2015). Capture Hi-C identifies the chromatin interactome of colorectal cancer risk loci. *Nat. Commun.* 6:6178. 10.1038/ncomms7178 25695508 PMC4346635

[B20] Jeng M. Y. Mumbach M. R. Granja J. M. Satpathy A. T. Chang H. Y. Chang A. L. S. (2019). Enhancer connectome nominates target genes of inherited risk variants from inflammatory skin disorders. *J. Invest. Dermatol.* 139 605–614. 10.1016/j.jid.2018.09.011 30315781

[B21] Kelemen L. E. Lawrenson K. Tyrer J. Li Q. Lee J. M. Seo J. H. (2015). Genome-wide significant risk associations for mucinous ovarian carcinoma. *Nat. Genet.* 47 888–897. 10.1038/ng.3336 26075790 PMC4520768

[B22] King E. R. Tung C. S. Tsang Y. T. Zu Z. Lok G. T. Deavers M. T. (2011). The anterior gradient homolog 3 (AGR3) gene is associated with differentiation and survival in ovarian cancer. *Am. J. Surg. Pathol.* 35 904–912. 10.1097/PAS.0b013e318212ae22 21451362 PMC3095702

[B23] Kuchenbaecker K. B. Ramus S. J. Tyrer J. Lee A. Shen H. C. Beesley J. (2015). Identification of six new susceptibility loci for invasive epithelial ovarian cancer. *Nat. Genet.* 47 164–171. 10.1038/ng.3185 25581431 PMC4445140

[B24] Lancho O. Herranz D. (2018). The MYC enhancer-ome: long-range transcriptional regulation of MYC in cancer. *Trends Cancer* 4 810–822. 10.1016/j.trecan.2018.10.003 30470303 PMC6260942

[B25] Li L. Barth N. K. H. Pilarsky C. Taher L. (2019). Cancer is associated with alterations in the three-dimensional organization of the genome. *Cancers* 11:1886. 10.3390/cancers11121886 31783642 PMC6966451

[B26] Li Y. Haarhuis J. H. I. Sedeno Cacciatore A. Oldenkamp R. van Ruiten M. S. Willems L. (2020). The structural basis for cohesin-CTCF-anchored loops. *Nature* 578 472–476. 10.1038/s41586-019-1910-z 31905366 PMC7035113

[B27] Li Y. Sun J. Gao S. Hu H. Xie P. (2018). HOXB4 knockdown enhances the cytotoxic effect of paclitaxel and cisplatin by downregulating ABC transporters in ovarian cancer cells. *Gene* 663 9–16. 10.1016/j.gene.2018.04.033 29660518

[B28] Lichtenstein P. Holm N. V. Verkasalo P. K. Iliadou A. Kaprio J. Koskenvuo M. (2000). Environmental and heritable factors in the causation of cancer–analyses of cohorts of twins from Sweden, Denmark, and Finland. *N. Engl. J. Med.* 343 78–85. 10.1056/NEJM200007133430201 10891514

[B29] Lopez-Isac E. Acosta-Herrera M. Kerick M. Assassi S. Satpathy A. T. Granja J. (2019). GWAS for systemic sclerosis identifies multiple risk loci and highlights fibrotic and vasculopathy pathways. *Nat. Commun.* 10:4955. 10.1038/s41467-019-12760-y 31672989 PMC6823490

[B30] Matulonis U. A. Sood A. K. Fallowfield L. Howitt B. E. Sehouli J. Karlan B. Y. (2016). Ovarian cancer. *Nat. Rev. Dis. Primers* 2:16061. 10.1038/nrdp.2016.61 27558151 PMC7290868

[B31] Miguel-Escalada I. Bonas-Guarch S. Cebola I. Ponsa-Cobas J. Mendieta-Esteban J. Atla G. (2019). Human pancreatic islet three-dimensional chromatin architecture provides insights into the genetics of type 2 diabetes. *Nat. Genet.* 51 1137–1148. 10.1038/s41588-019-0457-0 31253982 PMC6640048

[B32] Miller K. R. Patel J. N. Zhang Q. Norris E. J. Symanowski J. Michener C. (2018). HOXA4/HOXB3 gene expression signature as a biomarker of recurrence in patients with high-grade serous ovarian cancer following primary cytoreductive surgery and first-line adjuvant chemotherapy. *Gynecol. Oncol.* 149 155–162. 10.1016/j.ygyno.2018.01.022 29402501

[B33] Mok S. C. Bonome T. Vathipadiekal V. Bell A. Johnson M. E. Wong K. K. (2009). A gene signature predictive for outcome in advanced ovarian cancer identifies a survival factor: microfibril-associated glycoprotein 2. *Cancer Cell* 16 521–532. 10.1016/j.ccr.2009.10.018 19962670 PMC3008560

[B34] Montefiori L. E. Sobreira D. R. Sakabe N. J. Aneas I. Joslin A. C. Hansen G. T. (2018). A promoter interaction map for cardiovascular disease genetics. *eLife* 7:e35788. 10.7554/eLife.35788.103 PMC6053306 29988018

[B35] Monterisi S. Riso P. Lo Russo K. Bertalot G. Vecchi M. Testa G. (2018). HOXB7 overexpression in lung cancer is a hallmark of acquired stem-like phenotype. *Oncogene* 37 3575–3588. 10.1038/s41388-018-0229-9 29576613

[B36] Mumbach M. R. Rubin A. J. Flynn R. A. Dai C. Khavari P. A. Greenleaf W. J. (2016). HiChIP: efficient and sensitive analysis of protein-directed genome architecture. *Nat. Methods* 13 919–922. 10.1038/nmeth.3999 27643841 PMC5501173

[B37] O’Mara T. A. Spurdle A. B. Glubb D. M. Endometrial C. (2019). Cancer association, analysis of promoter-associated chromatin interactions reveals biologically relevant candidate target genes at endometrial cancer risk loci. *Cancers* 11:1440. 10.3390/cancers11101440 31561579 PMC6826789

[B38] Permuth-Wey J. Lawrenson K. Shen H. C. Velkova A. Tyrer J. P. Chen Z. (2013). Identification and molecular characterization of a new ovarian cancer susceptibility locus at 17q21.31. *Nat. Commun.* 4:1627. 10.1038/ncomms2613 PMC3709460 23535648

[B39] Pharoah P. D. Tsai Y. Y. Ramus S. J. Phelan C. M. Goode E. L. Lawrenson K. (2013). GWAS meta-analysis and replication identifies three new susceptibility loci for ovarian cancer. *Nat. Genet.* 45 362–370, 370e1–e2. 10.1038/ng.2564 23535730 PMC3693183

[B40] Phelan C. M. Kuchenbaecker K. B. Tyrer J. P. Kar S. P. Lawrenson K. Winham S. J. (2017). Identification of 12 new susceptibility loci for different histotypes of epithelial ovarian cancer. *Nat. Genet.* 49 680–691. 10.1038/ng.3826 28346442 PMC5612337

[B41] Pomerantz M. M. Ahmadiyeh N. Jia L. Herman P. Verzi M. P. Doddapaneni H. (2009). The 8q24 cancer risk variant rs6983267 shows long-range interaction with MYC in colorectal cancer. *Nat. Genet.* 41 882–884. 10.1038/ng.403 19561607 PMC2763485

[B42] Schmitt A. D. Hu M. Jung I. Xu Z. Qiu Y. Tan C. L. (2016). A compendium of chromatin contact maps reveals spatially active regions in the human genome. *Cell Rep.* 17 2042–2059. 10.1016/j.celrep.2016.10.061 27851967 PMC5478386

[B43] Servant N. Varoquaux N. Lajoie B. R. Viara E. Chen C. J. Vert J. P. (2015). HiC-Pro: an optimized and flexible pipeline for Hi-C data processing. *Genome Biol.* 16:259. 10.1186/s13059-015-0831-x 26619908 PMC4665391

[B44] Song H. Ramus S. J. Tyrer J. Bolton K. L. Gentry-Maharaj A. Wozniak E. (2009). A genome-wide association study identifies a new ovarian cancer susceptibility locus on 9p22.2. *Nat. Genet.* 41 996–1000. 10.1038/ng.424 19648919 PMC2844110

[B45] Sotelo J. Esposito D. Duhagon M. A. Banfield K. Mehalko J. Liao H. (2010). Long-range enhancers on 8q24 regulate c-Myc. *Proc. Natl. Acad. Sci. U.S.A.* 107 3001–3005. 10.1073/pnas.0906067107 20133699 PMC2840341

[B46] Stavnes H. T. Holth A. Don T. Kaern J. Vaksman O. Reich R. (2013). HOXB8 expression in ovarian serous carcinoma effusions is associated with shorter survival. *Gynecol. Oncol.* 129 358–363. 10.1016/j.ygyno.2013.02.021 23438671

[B47] Taberlay P. C. Achinger-Kawecka J. Lun A. T. Buske F. A. Sabir K. Gould C. M. (2016). Three-dimensional disorganization of the cancer genome occurs coincident with long-range genetic and epigenetic alterations. *Genome Res.* 26 719–731. 10.1101/gr.201517.115 27053337 PMC4889976

[B48] Yeung T. L. Leung C. S. Wong K. K. Gutierrez-Hartmann A. Kwong J. Gershenson D. M. (2017). ELF3 is a negative regulator of epithelial-mesenchymal transition in ovarian cancer cells. *Oncotarget* 8 16951–16963. 10.18632/oncotarget.15208 28199976 PMC5370013

[B49] Ying Y. Wang Y. Huang X. Sun Y. Zhang J. Li M. (2020). Oncogenic HOXB8 is driven by MYC-regulated super-enhancer and potentiates colorectal cancer invasiveness via BACH1. *Oncogene* 39 1004–1017. 10.1038/s41388-019-1013-1 31591481

[B50] Yu H. Y. Pan S. S. (2020). MiR-202-5p suppressed cell proliferation, migration and invasion in ovarian cancer via regulating HOXB2. *Eur. Rev. Med. Pharmacol. Sci.* 24 2256–2263. 32196576 10.26355/eurrev_202003_20491

