# Supplementary material for: Functional Interrogation of Enhancer Connectome Prioritizes Candidate Target Genes at Ovarian Cancer Susceptibility Loci
Source: Front Genet. 2021 Mar 19;12:646179. doi: 10.3389/fgene.2021.646179 (PMC8017555; doi:10.3389/fgene.2021.646179)
Supplement: Supplementary Figure 1 — QC of HiChIP results with HiC-pro pipeline and loop calling results with Fit-HiChIP pipeline. [file Data_Sheet_1.docx]

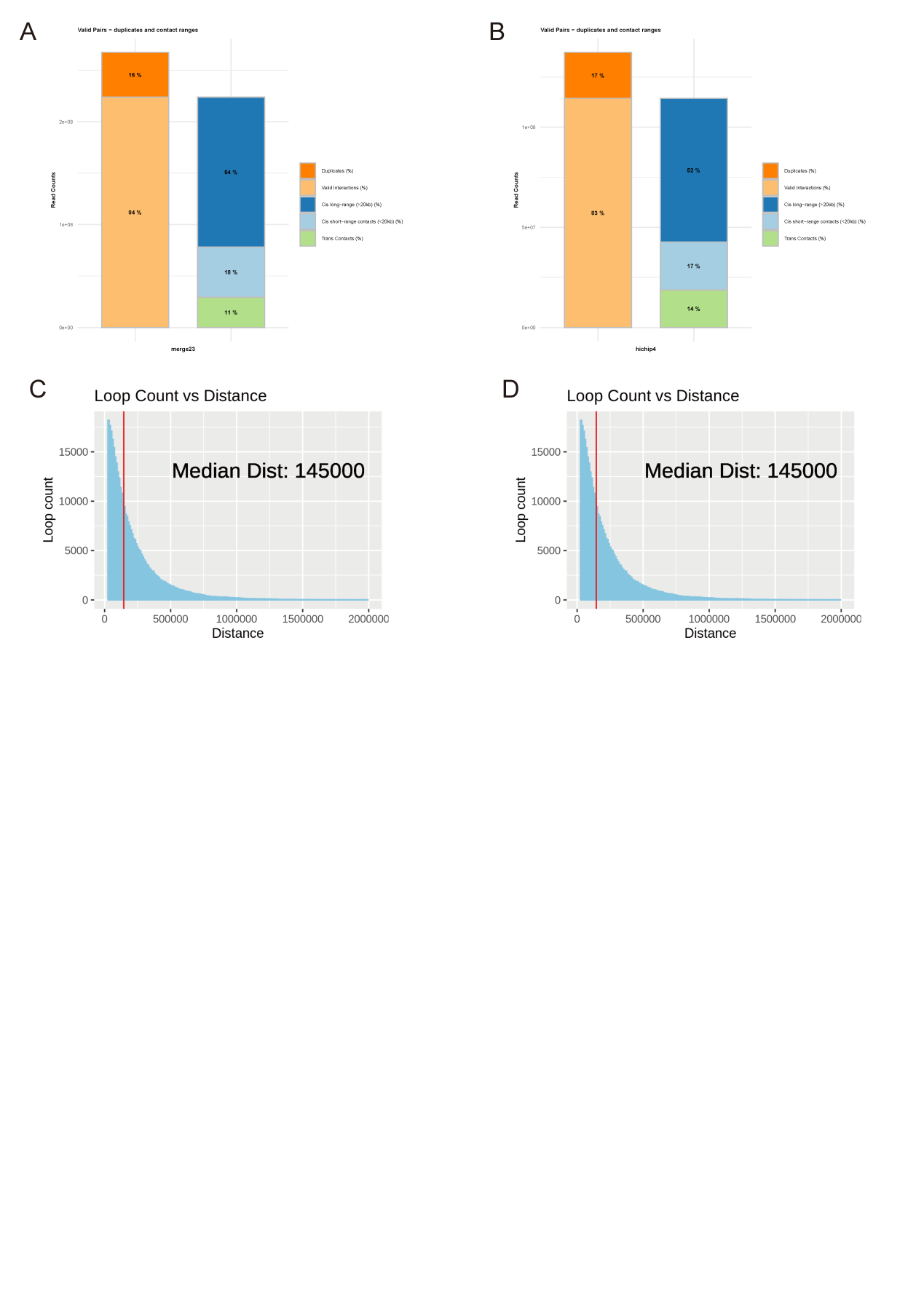


**Supplementary figure 1. QC of HiChIP results with HiC-pro pipeline and Loop calling results with Fit-HiChIP pipeline.** (A, B)QC results of HiChIP library generated with SKOV3 (A) and OVCA432 (B) cells. The fractions of duplicated reads, as well as short range versus long range interactions were reported. (C, D)Loop-calling results from Fit-HiChIP pipeline for SKOV3 (C) and OVCA432 (D) cells. Loop counts versus distance were reported. Red line depicts the median distance for the significant loops.


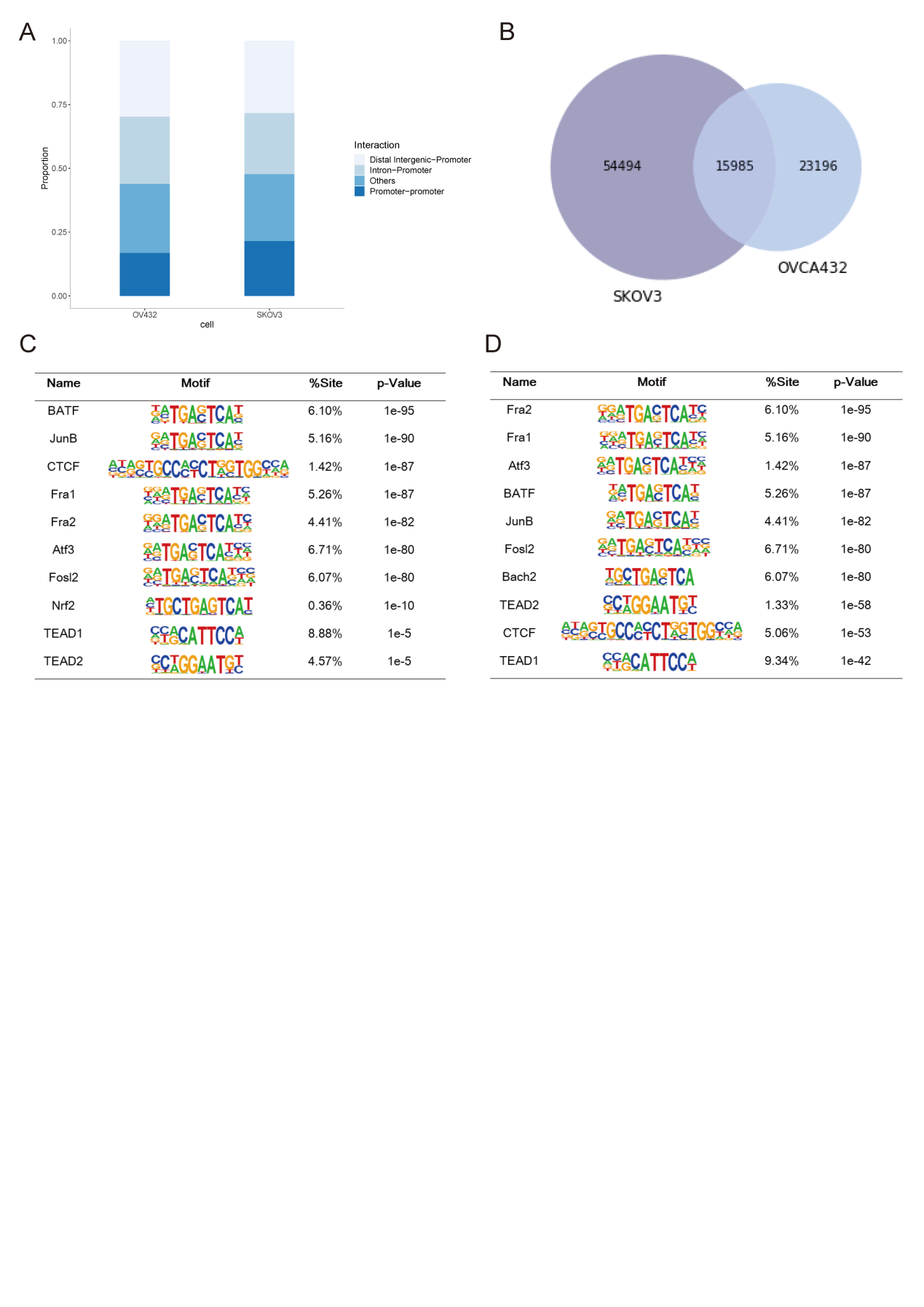


**Supplementary figure 2. General features of Fit-HiChIP identified loops in EOC.** (A) Proportion of promoter-associated interactions in each category in SKOV3 and OVCA432 cells: Distal intergenic – promoter, Intron – promoter, Others (3’UTR, 5’UTR, exon, downstream, upstream) – promoter, Promoter – promoter. (B) Venn diagram displaying the number of cell-specific and shared significant loops in SKOV3 and OVCA432. (C, D) Transcription factor motif enrichment in non-promoter end of promoter-associated loops. Top ranked transcription factor (TF) motifs identified using HOMER in the ATAC-seq identified open chromatin region from non-promoter end of promoter-associated loops in SKOV3 (C) and OVCA432 (D).


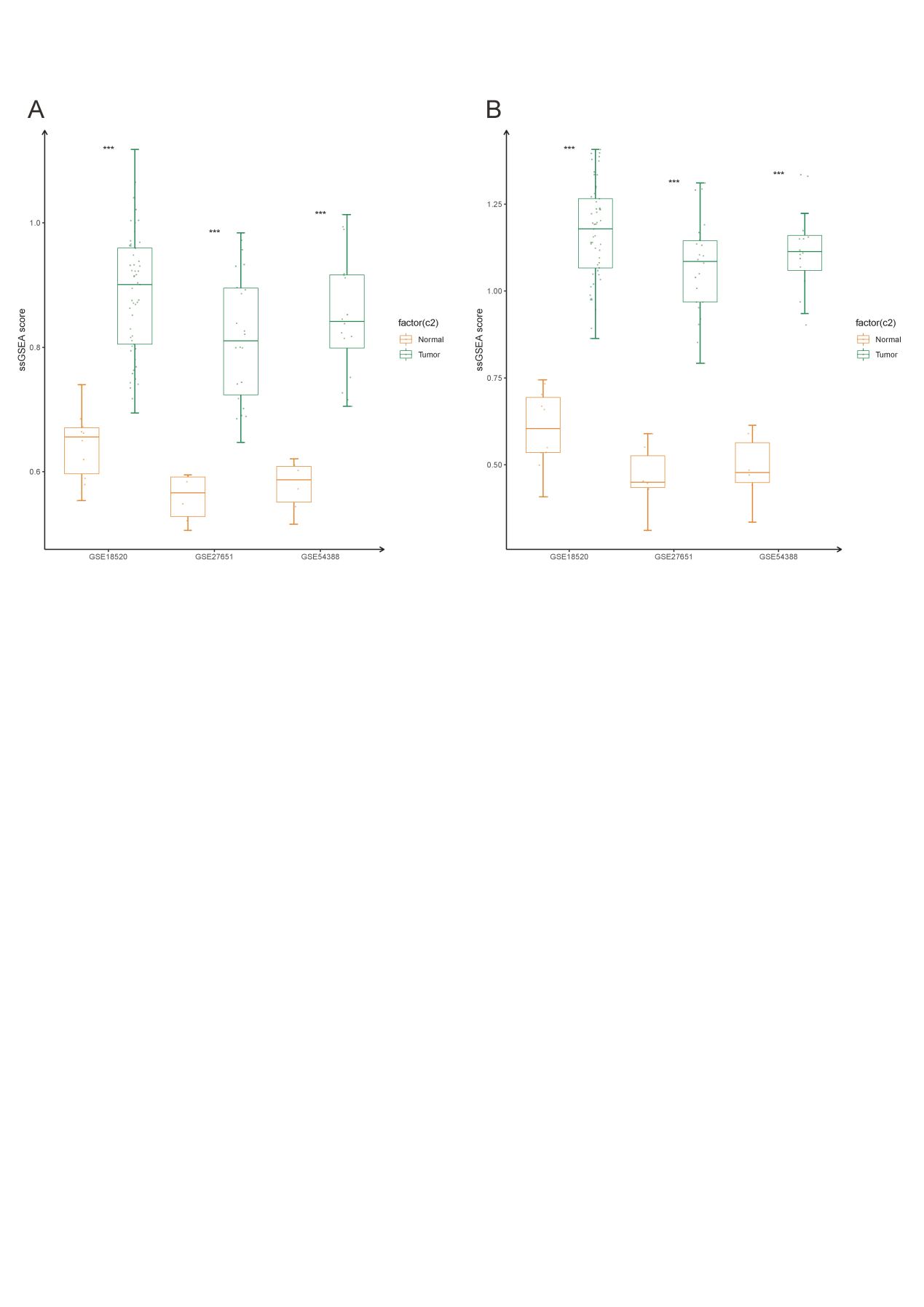


**Supplementary figure 3. Enrichment score of HiChIP target genes calculated by ssGSEA between normal and tumor ovarian samples in three GEO datasets. (A, B)**The enrichment score of 162 HiChIP target genes (A) and 39 differentially expressed HiChIP target genes (B) was estimated and plotted for each normal or tumor ovarian sample of three GEO datasets. The middle line in the box indicates median and the bound indicates 25% quartile (Q1) and 75% quartile (Q3). ***p < 0.001 as determined by an unpaired, two-tailed Student’s t test.





**Supplementary figure 4. Expression of SKAP1 expression in SKOV3 and OVCA432 cells after modulating rs9303542 enhancer region.** (A, B) qPCR was used to detect the expression of SKAP1 between rs9303542 deleted (DEL) and vector control (EV) cells in SKOV3(A) and in OVCA432(B). (C) qPCR was used to compare the expression of SKAP1 after sgRNA1/2 transfected (sgRNA1, sgRNA2) and empty vector transfected (EV) cells with dCas9-VP64 stable expression. ns: not significant, as determined by an unpaired, two-tailed Student’s t test.


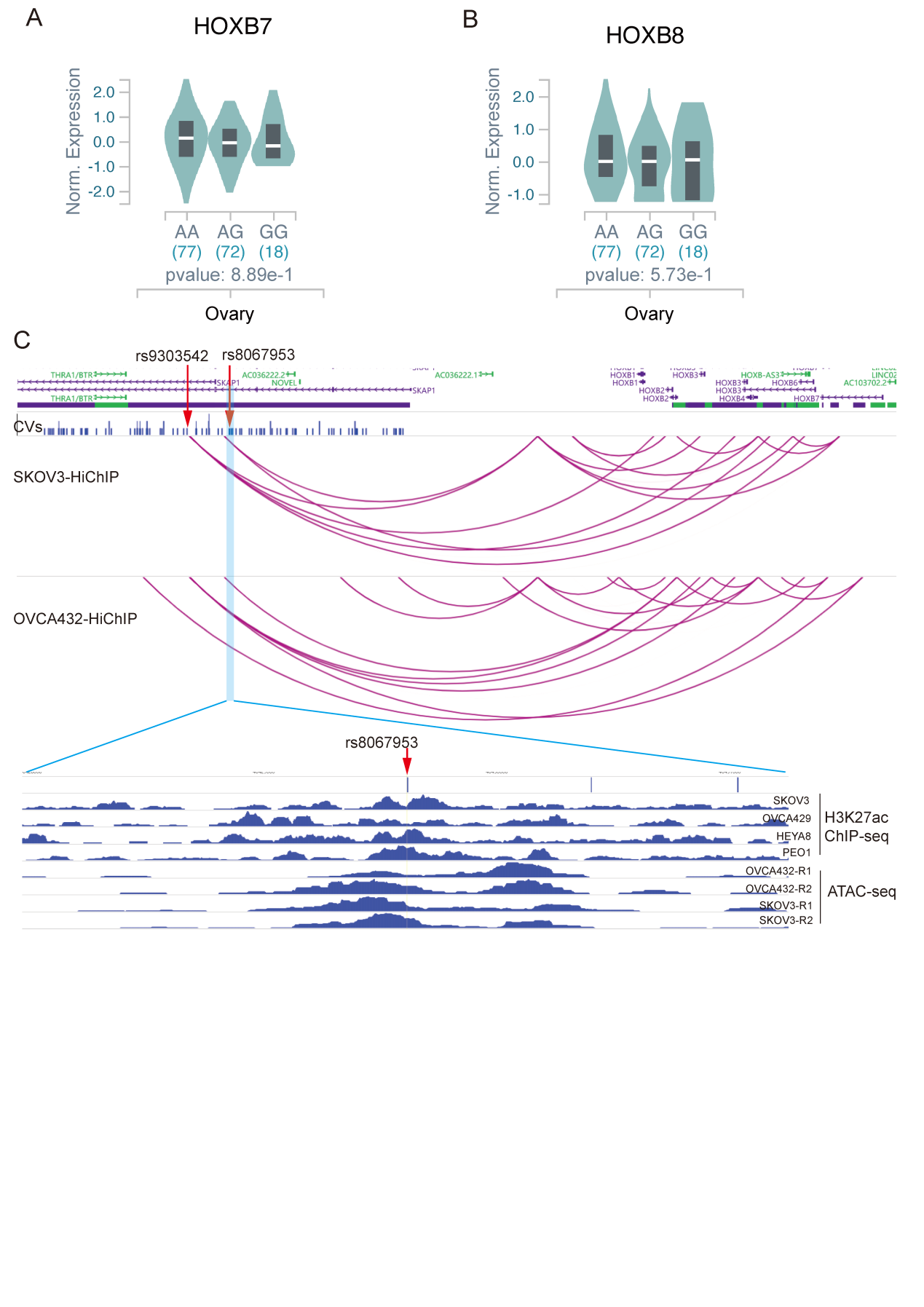


**Supplementary figure 5. eQTL analysis of HOXB7 and HOXB8 as well as loop profiles of rs8067953.** (A, B) eQTL analysis for HOXB7 (A) and HOXB8 (B) analysis with ovary tissue samples from GTEx database. (C) Interaction profiles of rs8067953 and HOXB genes at 17q21.32 and ATAC-seq and H3K27ac-ChIP signal enrichment at the rs8067953 region. (D).
